# Development and Validation of a Multidimensional Nursing Empowerment Scale (NES)

**DOI:** 10.1155/jonm/5594415

**Published:** 2026-08-30

**Authors:** Fatma Cerit Soydan, Ayşe Nefise Bahçecik

**Affiliations:** ^1^ Health Sciences Faculty, Department of Nursing, Istanbul Topkapı University, Istanbul, Turkey; ^2^ Health Sciences Faculty, Department of Nursing, Istanbul Sabahattin Zaim University, Istanbul, Turkey, iszu.edu.tr

**Keywords:** empowerment, nursing, reliability and validity, scale development

## Abstract

**Background:**

Empowerment in nurses is a multidimensional construct that has favorable results for both the patient and the employee and is emphasized in order to provide high‐quality healthcare. For this concept to be translated into practice, measurement tools developed in this field are of critical importance. Although several validated instruments assess structural, psychological, or behavioral empowerment separately, each having been validated independently, using distinct samples, scoring frameworks, and item structures, no instrument evaluating all three dimensions simultaneously within a single scale has been identified. This gap limits nurse managers in comprehensively diagnosing empowerment and in evaluating the effectiveness of empowerment interventions.

**Objective:**

The study was conducted to develop a valid and reliable measurement tool aiming to determine the empowerment levels of nurses in a multidimensional manner in terms of structural, psychological, and behavioral dimensions.

**Methods:**

In this methodological study, the standard scale development and validation procedures required for instruments to be psychometrically sound were employed. An initial 256‐item pool was generated based on a comprehensive literature review and an autoethnographic approach involving 36 nurses. Subsequently, face and content validity (Lawshe’s technique) were evaluated. Data were collected between May and September 2017 from nurses (*N* = 1992) working across 10 hospitals affiliated with a regional health group in Istanbul; the study was completed with a final sample of 743 nurses (37.3%). Exploratory factor analysis (EFA) and confirmatory factor analysis (CFA) were used to determine the construct validity of the scale. Cronbach’s alpha, item analyses, and test–retest analyses were employed to assess reliability.

**Results:**

The draft nursing empowerment scale (NES), developed as a result of the face and content validity studies, consisted of 136 items and was administered to the sample group. The EFA conducted resulted in a NES consisting of 103 items and three factors (structural, psychological, and behavioral), explaining 49.95% of the total variance (KMO = 0.97; Bartlett *χ* ^2^ = 58,144.65, *p* < 0.001). The scale factor structure was supported by supplementary CFA. The goodness‐of‐fit indicators (*χ* ^2^/df = 3.75; RMSEA = 0.06; CFI = 0.92; NFI = 0.90; GFI = 0.90; AGFI = 0.88) were favorable. Internal consistency was observed (Cronbach’s *α* = 0.98 overall; subscale *α* = 0.91–0.98) and 15‐day test–retest correlation was *r* = 0.86 (subscale *r* = 0.73–0.83).

**Conclusions:**

The NES demonstrates good psychometric properties as a comprehensive, multidimensional research instrument. Future research should test the scale on independent and diverse samples. Its considerable length (103 items) is acknowledged as a practical limitation, and the development of a shorter, practice‐oriented form is planned.

**Implications for Nursing Management:**

The NES enables the simultaneous, multidimensional measurement of structural, psychological, and behavioral empowerment within a single instrument. It could be useful in determining empowerment levels and evaluating the effectiveness of targeted empowerment strategies.

## 1. Introduction

Empowerment has become one of the most strategically discussed concepts in contemporary nursing management. The persistent global nursing shortage and workforce mobility make retaining and empowering nurses a priority. Nurses in empowered environments tend to stay in their jobs, and adequate staffing and education significantly reduce patient mortality [1, 2]. Furthermore, inadequate nurse staffing has been shown to adversely affect the quality of patient–nurse communication [3], increase the risk of sickness absence among nurses, and pose substantial risks to patient safety [4]. Nurses, who make up the majority (59%) of healthcare workers and have direct contact with patients, are of critical clinical importance in ensuring patient‐centered and cost‐efficient care [5, 6].

Empowerment has been shown to increase professional job satisfaction, organizational trust, unit effectiveness, and positive collegial relationships [7] and to strengthen the culture of patient safety [8, 9], thereby exerting a favorable impact on healthcare delivery [10]. Asif et al. reported that empowerment reduces adverse patient‐related outcomes and improves the quality of patient care [11]. Moreover, as nurses’ levels of psychological empowerment increase, their caring behaviors also improve [12].

The heavy workload, stress, exhaustion, and illnesses resulting in missed days and resignations have a negative impact on nurses’ own health as well as patients and the organization as a whole. Empowerment is a noted concept in coping with these negative situations, in increasing job satisfaction and performance [10, 12–15] and also in decreasing occupational exhaustion [16–19].

In this context, the need to make nurses more effective and empower them to achieve 21st‐century healthcare objectives has been highlighted [20]. In line with this, the International Council of Nurses (ICN) reiterated the global importance of empowerment by designating the theme for International Nurses Day 2026 as “Our Nurses, Our Future: Empowered Nurses Save Lives” [21].

### 1.1. Theoretical Framework

Empowerment is defined as “a personal development process improving the decision‐making rights and requests of individuals through mutual help, nurturing, sharing, and teamwork” [16]. The empowerment concept is evaluated through various points of view in the literature.

The foundation of the organizational/structural theories that explain empowerment with factors outside the employees is the “Theory of Structural Empowerment in Organizations” by Kanter. The objective of this theory is to determine the structural elements that prevent participation of the employees in the organization and make the employees passive and powerless and to revise these elements [22–24]. According to Kanter, power is “the capacity to mobilize resources to accomplish a task.” Within organizations, there are structural conditions (opportunity, information, support, and resources) and opportunity structures that provide access to growth and development. These structures are influenced by the degree of formal and informal power held by the individual within the organizational structure [25].

The first descriptive studies on psychological empowerment were conducted by Conger and Kanungo [26]. They defined empowerment as “the process of increasing belief in self‐adequacy.” Following this definition, psychological empowerment has been expanded and conceptualized as intrinsic task motivation by Thomas and Velthouse [27] and Spreitzer [28]. Psychological empowerment is now defined as the perceptions and tendencies of the employee regarding his/her organizational role and work [29]. Reflecting an active orientation to a work role and stemming from a sense of control, psychological empowerment is a multidimensional task motivation. This active cognitive state consists of the combination of four dimensions. These dimensions are as follows: the belief that one’s work is important (meaning); the belief in one’s capability to perform the job (competence); having autonomy in carrying out one’s role (self‐determination); and the belief that one’s activities influence outcomes (impact) [30].

Psychological empowerment is associated with work‐related perceptions but does not mention the concrete behavior used to affect this work. However, Irvine et al. [31] conducted a study describing and measuring empowerment using concrete behavior. This study sparked interest in behavioral empowerment (BE), which characterizes empowerment both as behaviors inherent to the employee’s role and as behaviors that extend beyond it. Through this point of view, empowerment was defined as a process that enables individuals to trust in their own capability to carry out a specific action successfully [32]. BE is defined as a three‐factor structure comprising verbal behaviors (expressing and discussing opinions and suggestions), behavioral actions (identifying problems, gathering information, proposing solutions, acquiring new skills, and performing more challenging tasks, both as a group and individually), and outcome‐oriented behaviors (identifying and resolving the causes of problems, improving the way work is done, and enhancing organizational effectiveness) [33].

These three perspectives are conceptually sequential rather than independent: structural conditions shape how employees perceive their organizational role, which is expressed as psychological empowerment [26, 27, 30]. Spreitzer [30] and Menon [34] both argue that structural and psychological empowerment are not independent constructs but jointly constitute the empowerment process. A meta‐analysis on this topic confirms that structural and psychological empowerment are interrelated components of a single underlying construct [24]. Psychological empowerment is enacted as observable empowered behavior [31]. This sequential, integrative logic underlies the development of the nursing empowerment scale (NES).

### 1.2. Measuring Empowerment in Nursing

Several validated instruments have been developed to measure empowerment in nursing. Existing instruments generally capture a single dimension of empowerment. Structural empowerment is most often measured with the Conditions of Work Effectiveness Questionnaire (CWEQ‐II) [35], the Practice Environment Scale of the Nursing Work Index [36], the Empowering Management Practices Scale (SEMP) used by Boudrias et al. [37], and the Social‐Structural Characteristics Scale developed by Spreitzer [28].

Psychological empowerment is assessed with Spreitzer’s Psychological Empowerment Instrument (PEI) [30] or Roller’s Perception of Empowerment Instrument [38], and has also been evaluated using The Empowerment Scale developed by Menon [34] and the Qualities of an Empowered Nurse Scale (QEN‐S) developed by Kuokkanen et al. [39].

A review of the literature reveals that BE has also been measured using the Work Empowerment Scale [31], the BE Questionnaire [37], and the Performance of Empowered Nurse Scale (PEN‐S) developed by Kuokkanen et al. [39].

Achieving empowerment in practice remains challenging despite its widespread use in the nursing literature [23]. A review of the empowerment studies in Turkey reports that the current studies on empowerment in nursing are inadequate and further studies are needed [40]. O’Grady and Clavelle [41] similarly emphasize the need for multidimensional evaluation to comprehend empowerment and transfer the results into practice.

As noted above, studies examining empowerment in nursing are required. Existing empowerment scales have each made valuable contributions to our understanding of structural, psychological, and BE, typically by focusing in depth on one of these constructs. These instruments, however, were developed and validated independently, each within its own sample and psychometric framework. As a result, researchers seeking a comprehensive assessment of empowerment must administer multiple tools and then contend with the challenge of integrating and correlating scores across instruments that were never validated together in the first place.

To address this gap, the proposed NES validates all dimensions simultaneously within a unified psychometric framework, yielding empirically interrelated subdimension scores alongside a composite overall score. The NES serves as an *umbrella* instrument for holistic evaluation. Consequently, this scale is anticipated to contribute significantly to nursing practice and healthcare administration.

### 1.3. Aim

The current study aimed to:

1. Develop a multidimensional NES enabling the evaluation of nurses’ empowerment levels in terms of structural, psychological, and behavioral dimensions.
2. Conduct the validity and reliability studies of the developed scale.

## 2. Materials and Methods

### 2.1. Research Design

**FIGURE 1:**
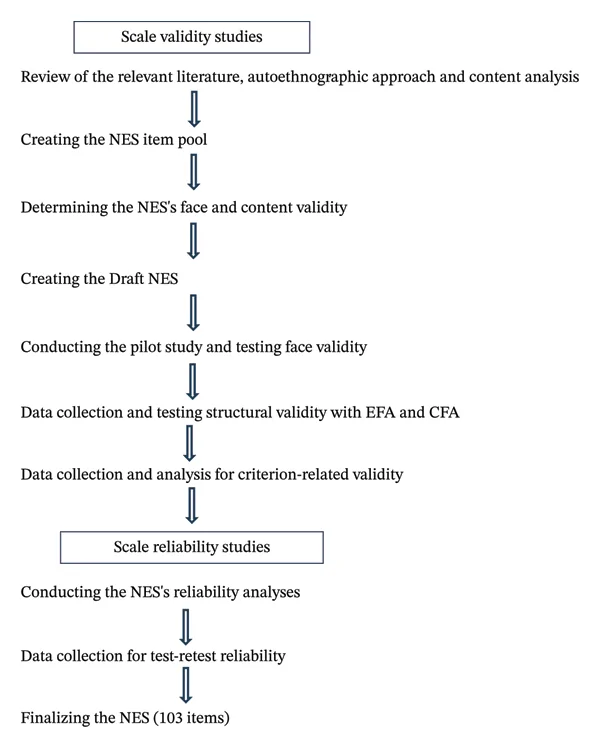
Stages of scale development.

This methodological study was conducted in accordance with the scale development and validation process [42, 43]. The steps followed in the scale development and validation process of the Empowerment Scale are presented in Figure 1.

### 2.2. Creation of the Item Pool for the Measurement Tool

A literature review, autoethnographic approach and content analyses were used in the creation of the scale’s item pool. An attempt was made to keep the item pool, created to develop a single scale with a holistic approach to empowerment, large so that it could be multidimensional. A literature search was conducted using the “empowerment”, “personnel empowerment”, “nursing empowerment” in various databases in order to define the conceptual structure related to empowerment. A literature survey showed that the studies were usually related to the structural, psychological, and BE areas. A total of 36 nurses working in various fields (13 clinical nurses, 10 managers, 6 academicians, and 7 special branch nurses) were individually interviewed using semi‐structured interviews in order to obtain their views on empowerment. In qualitative studies, a sample size of 5–25 participants is suggested for phenomenological studies, while 20–30 participants is recommended for grounded theory [44]. In this context, the group of 36 participants was considered sufficient to ensure adequate diversity. They were asked open‐ended questions about empowerment (e.g., What workplace characteristics make you feel empowered? How do you behave when you feel empowered?) and encouraged to express their views freely. Data obtained through the semi‐structured interview technique were evaluated using content analysis (without structured coding) and grouped under the themes of structural, psychological, and BE. Responses were analyzed through content analysis together with the literature review to generate candidate items. The conceptual principles of Kanter’s Theory were used as the basis for developing the item pool defining structural empowerment. The current measurement tools on the subject [28, 35, 36, 45] were reviewed. The item pool for psychological empowerment was informed by the self‐efficacy concept introduced by Conger and Kanungo [26] and further developed into the cognitive motivational model of Thomas and Velthouse [27], which addresses how empowerment is perceived and interpreted by employees. The relevant measurement tools [34, 38, 39] were reviewed. During the development of scale items related to BE, the point of view was based on the cognitive model stating that empowerment is feeling sure that a particular behavior can be displayed and maintained. The measurement tools developed within this context [31, 37, 39] were reviewed. Following the literature review and autoethnographic approach, an item pool that would help determine the empowerment levels of the nurses was created. Sample items for structural empowerment are “Employee rights are respected”, “Employees act as a part of the whole”, “Employees have the necessary training and learning opportunities”, “The staff number is adequate”, “The task definitions are clearly defined”, “An attempt is made to be fair”. Sample items of the psychological empowerment included “I am not an effective worker at my workplace”, “I believe I can make a difference at my workplace”, “I have the necessary skills to do my job successfully”, “It is important for me to do my job well”, “I feel the responsibility of the work I do”, “I frequently think that what I do or feel regarding my work makes no difference”. BE items encompass empowered nursing behaviors, such as “I try to cope with the difficulties associated with my work”, “I frequently ask myself how I can do my job better”, “I can easily defend my opinions at the workplace”, “I convey any problems to my managers without any thinking”, “I participate in group work”, “I try to improve my work”.

The measurement type was determined as a 5‐point Likert‐type scale (1 = “definitely disagree,” 2 = “disagree,” 3 = “undecided,” 4 = “agree,” 5 = “definitely agree”). Scoring was performed by adding all the item scores for a subdimension and the whole scale and then dividing the result by the number of items. Accordingly, possible scores for each subdimension and for the overall scale range from 1.00 to 5.00. Increased scores were accepted to indicate increased empowerment perception in the relevant subdimension and the total score. There is no cutoff point of the scale, and a norm study has not been conducted for descriptive classification of the scale score, such as low, moderate, or high. However, when categorization is specifically required, the scale score range may be divided into three equal intervals. Accordingly, a mean score of *x̄* < .33 may be interpreted as “low,” scores between *x̄* = 2.33 and 3.66 as “moderate,” and scores of *x̄* ≥ 3.66 as “high.”

### 2.3. Obtaining Expert Opinions for the Items

The NES item pool was submitted for expert review to evaluate its face and content validity. It has been reported that obtaining expert opinions from 5 to 40 specialists increases scale validity by ensuring that items are easy to understand, suitable for their intended purpose, and appropriate in terms of response format [46, 47].

#### 2.3.1. Face Validity

The items in the nursing empowerment item pool were reviewed by a Turkish language specialist. The necessary revisions were made so that the items would be appropriate for the language and could be understood.

The face validity of the created initial item pool was evaluated by 17 specialists in the field with at least a Ph.D. degree (nursing management, psychiatric nursing, fundamentals of nursing, public health nursing, management and organization, and measurement and evaluation). Face and content validity assessments were conducted concurrently. The field specialists were asked to evaluate the items in the nursing empowerment item pool for their suitability regarding the language, how easy they were to understand, their similarity with each other, their ability to explain the concept, the suitability for the sample audience, and the conformity of the items. In addition, their opinions were obtained with open‐ended questions. The researchers refined the item pool based on the evaluations provided by the field experts.

#### 2.3.2. Content Validity

The field experts evaluated content validity subsequent to the evaluation of face validity. The Lawshe technique (CVR) was applied using item‐level ratings from 15 of the 17 experts (2 experts gave only overall, nonitem‐level ratings and were excluded from the CVR calculation). The specialists classified item suitability as “suitable,” “suitable but inadequate,” or “not suitable”. With the Lawshe technique, the minimum content validity ratio for the 15 specialists was accepted as 0.49 [46]; items with a lower value were removed from the item pool, and the recommended revisions were made.

It is recommended that the revised scale form be administered to a small group prior to the main sample in order to evaluate the clarity of the instructions and items, as well as the adequacy of the administration setting and duration [42]. Preliminary application of the draft NES was performed on a group of 41 nurses in this respect. The suitability of the scale to the sample group and the applicability were tested. The 41‐nurse pilot assessed the comprehensibility of instructions/items and administration setting/duration. This group was excluded from the sample group.

### 2.4. Data Collection Tools

A Nurse Descriptive Information Form and the draft NES were used for data collection. The Descriptive Information form consisted of questions on the personal and occupational characteristics (age, gender, education, employment duration, etc.) of the nurses.

The CWEQ II [35] and the PEI [30] were used to determine the criterion validity of the structural and psychological empowerment subdimensions. The Turkish versions of both instruments were used [48]. However, no suitable Turkish instrument was available for assessing BE. These two instruments were also administered to a separate subsample of 58 nurses (not included in the main 743‐nurse sample) to assess criterion validity.

### 2.5. Administering the Scale to the Sample Group and Data Collection

Following the preliminary application, the draft NES was administered to the sample group. The study universe consisted of nurses (*N* = 1992) working in 10 hospitals (3 training and research hospitals and 7 state hospitals) belonging to a regional hospital group in Istanbul that granted permission for the study. There was no sample selection in the study; total‐population sampling was targeted. All nurses who worked in these hospitals and accepted study participation were included in the study scope. The inclusion criterion was voluntary participation. Exclusion criteria comprised nonvolunteering or being on official leave or sick leave during the data‐collection period.

The data collection tools (draft NES and Descriptive Information Form) were administered with the face‐to‐face interview method between May 10 and September 2, 2017, after written consent was obtained. On average, participants took 15–20 min to complete the data collection tools. The data collection tools were handed over manually to the 912 nurses who accepted to join the study, and a total of 804 nurses completed the surveys and returned them. Surveys with incorrect or missing data or where there were items left empty in the survey section were eliminated from the study (7.6%). The study was completed with a final sample of 743 nurses. Exploratory factor analysis (EFA) and confirmatory factor analysis (CFA) were conducted on the same sample. The sample size adequacy was determined by using the relevant guidelines to ensure robust analyses for EFA and CFA [42, 49, 50].

Various values are proposed as to what the sample size should be in order to ensure strong results in factor analysis. For example, Hair et al. report that sample size can be calculated from the item number and should be at least 5 times larger [51]. The initial item number of 136 indicated that the sample size was adequate (136 × 5 = 680). In addition, Comrey and Lee [49] report 500–1000 subjects to be “very good” and 1000 to be “excellent” while Tabachnick and Fidell state that a sample size of 300 or more is adequate for EFA [52]. The sample size was assessed in line with these references.

### 2.6. Data Analysis

EFA was employed to determine the factor structure of the proposed NES [46]. The resulting structure was subsequently examined using CFA. Normality was assessed for the total scale score (*n* = 743; *M* = 3.35, SD = 0.61, min = 2, max = 5). Skewness (0.07) and kurtosis (0.19) values were examined to assess normality. In addition, the Kolmogorov–Smirnov test (0.032; *p* = 0.077) was used to determine whether the distributions met the assumption of normality within the sample [52]. The data were determined to come from a normal distribution [46, 53]. Pearson correlation analysis was utilized for item analyses, criterion‐related validity, and test–retest reliability. Dependent‐samples *t*‐tests were conducted to perform difference testing.

### 2.7. Conducting Validity and Reliability Studies for the Scale

The correlation matrix was evaluated to determine the suitability of the data for factor analysis prior to conducting the draft NES EFA, and items with *r* < 0.30 were removed from the pool. The Kaiser–Meyer–Olkin (KMO) measure was used to evaluate the adequacy of the sample size and the suitability of the data for factor analysis, while Bartlett’s test of sphericity was used to determine whether the data were derived from a multivariate normal distribution.

The principal component analysis (PCA) was used in determining the factor structures of the scale. PCA is one of the factoring methods used within the scope of EFA. PCA has been included in the process not as an independent analysis or as an alternative to EFA but as an analysis used within EFA procedures. PCA does not consider measurement errors, but confirmation was ensured with factor model fit indexes using CFA, which does consider such errors, at the end of the scale process. It was assumed that the subdimensions of the NES were associated with each other, and a decision was made to use ProMax analysis, one of the oblique rotation methods. Oblique rotation methods are used when it is assumed or determined that the factors within the measurement tool are associated with each other [54, 55]. The fact that the correlations between the factors were significant (*r*_min = 0.44, *r*_max = 0.69) confirms the appropriateness of this selection for this assumption. Promax rotation was preferred because it estimates factor correlations efficiently and offers faster, more practical computation compared with other oblique methods [56].

The factor load value lower cut‐off point was 0.30 for items to be included in the scale. An eigenvalue over 1 and the scree plot were used to determine the factor number [42]. Items with cross‐loading differences of ≤ 0.10 were removed during analyses. Factor analysis was continued using a fixed‐factor extraction procedure in order to theoretically justify the number of factors identified in the preceding analyses.

CFA was also used to examine the factor structure created following EFA. Chi‐square test statistics (*χ* ^2^), degrees of freedom (df), chi‐square/df (*χ* ^2^/df), root mean square error of approximation (RMSEA), normalized fit Index (NFI), comparative fit index (CFI), goodness of fit index (GFI), and the adjusted GFI (AGFI) were used to evaluate the goodness of fit of the model.

Due to the lack of a comprehensive, multidimensional empowerment scale, criterion‐related validity was analyzed at the subdimension level.

The Cronbach’s alpha coefficient was used to determine the NES’s internal consistency level. Item discrimination analyses and corrected item‐total correlation analyses were performed for item analysis. The test–retest reliability was assessed to evaluate the stability of the test over time. The sample size for the test–retest analysis was determined based on the Central Limit theorem [52]. The NES was administered to 53 nurses not included in the sample group, with an interval of 15 days (2 weeks).

### 2.8. Ethical Considerations

Before starting the study, permission was obtained from Marmara University’s Ethics Committee (Approval No. 81, 2016). The institutional permission was confirmed by the Regional Hospital Association of the hospitals where the study was conducted (Approval No. 774.99, 2017). Study‐related information was provided to all the nurses participating in the study, and their consent was obtained.

Participation was entirely voluntary, and participants were informed that they could withdraw from the study at any stage without any penalty or adverse consequences. Confidentiality and anonymity were maintained by using coded identifiers instead of personal information. The study was considered to involve minimal risk for the participants.

## 3. Results

### 3.1. Participant Characteristics

Among the 743 nurses who participated in the study, 87.9% (*n* = 653) were female, 59.8% (*n* = 444) were married, and 57.9% (*n* = 430) held a bachelor’s degree. The mean age of the nurses was 32.27 ± 7.79 years. Most participants worked as clinical nurses (76.9%, *n* = 571) in training‐and‐research hospitals (71.9%, *n* = 534). Regarding professional background, 40.0% (*n* = 297) had 3–10 years of overall nursing experience, while 50.2% (*n* = 373) had worked in their current unit for 2–6 years. The clinical units where the participants worked were most commonly medical (25.7%) and intensive care (24.7%) units, followed by surgical (18.6%), emergency (12.0%), specialty branch (11.3%), and operating room (7.7%) units (Table 1).

**TABLE 1:** Characteristics of the participants (*n* = 743).

Characteristics		*n*	%
Age	18–25	186	25.0
	26–33	253	34.1

Mean: 32.27 ± 7.79	34–41	202	27.2
	≥ 42	102	13.7

Gender	Female	653	87.9
	Male	90	12.1

Marital Status	Married	444	59.8
	Single	299	40.2

Educational Status	Health Vocational High School	89	12.0
	Associate Degree	118	15.9
	Bachelor’s Degree	430	57.9
	Postgraduate Education	106	14.2

Hospital of Employment	Training and Research Hospital	534	71.9
	State Hospital	209	28.1

Occupational Experience	≤ 2 years	143	19.2
	3–10 years	297	40.0
	11–18 years	141	19.0
	> 18 years	162	21.8

Position	Clinical Nurse	571	76.9
	Nurse Manager	88	11.8
	Nurse Specialist	84	11.3

Work Unit	Medical	191	25.7
	Surgery	138	18.6
	Emergency	89	12.0
	Intensive Care	184	24.7
	Special Branch Units^∗^	84	11.3
	Operating Room	57	7.7

Employment Duration at the Unit	≤ 1 year	223	30.0
	2–6 years	373	50.2
	7–11 years	99	13.3
	≥ 12 years	48	6.5

^∗^Diabetes nursing, quality nursing, organ transplantation nursing, education nursing, infection control nursing, and hemodialysis nursing.

### 3.2. Validity and Reliability Analyses Related to the Scale

#### 3.2.1. Content Validity

A total of 256 items (192 positively worded, 64 reverse‐worded) were generated through the literature review and semi‐structured interviews with nurses to enable the multidimensional assessment of nurses’ empowerment levels across the structural, psychological, and behavioral dimensions. As a result of the face validity evaluation, which included expert review for duplicate, similar, and unrelated items, the initial 256‐item pool was reduced to 159 items. Additionally, as a result of the content validity study, 23 items with a CVR below 0.49 were removed from the item pool. The pilot group verified the clarity, comprehensibility, and feasibility of the items within the designated duration. No further changes were made following the pilot application.

Following the completion of face and content validity assessments, the draft NES consisted of a total of 136 items, and the numbering was reorganized accordingly.

#### 3.2.2. Construct Validity Analyses

We excluded nine items exhibiting item‐total correlations below 0.30 from the pool prior to running the EFA on the draft NES. Factor analysis was continued with 127 items. The results yielded a KMO value of 0.97 and a significant Bartlett’s test of sphericity (*χ* ^2^ = 58,144.65, *p* < 0.001) confirming suitability for factor analysis.

**FIGURE 2:**
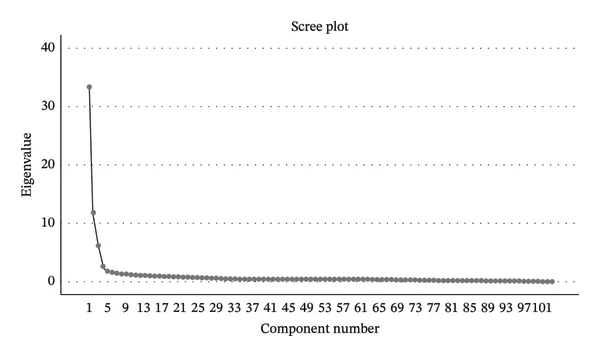
Factor analysis of the nursing empowerment scale‐scree plot.

In the first stage of the PCA, eight items with a loading value below 0.30 were removed from the item pool, and the analysis was continued with the remaining 119 items. Two‐stage factor analysis revealed 15 factors with an eigenvalue over 1.00. This number of factors could not be theoretically explained. The fixed factor extraction procedure was therefore used for the 3‐factor structure according to the theoretical structure that formed the initial basis of the scale development process. A total of 16 items were eliminated following the first stage rotation procedure, as they were cross‐loading items or items with low factor loadings. The procedure was continued with 103 items. Following the second stage rotation procedure, the NES consisted of 103 items with a three‐factor structure. The first factor consisted of 49 items, the second factor of 28 items, and the third factor of 26 items. Factor loads varied between 0.41 and 0.80. Among the scale items, 12 were inverse items with a negative load within the factor they were included in. As seen in the table and the scree‐plot distributions, the explained variance rate of the NES was found to be 49.95% (Table 2; Figure 2).

**TABLE 2:** Exploratory factor analysis results of the nursing empowerment scale.

Factor	Variance%	Number of items	Factor loadings
1st Factor	32.39	49	0.46–0.80
2nd Factor	11.56	28	0.49–0.79
3rd Factor	6.01	26	0.41–0.75
Total (GES)	49.95	103	0.41–0.80

CFA was employed to test the three‐factor, 103‐item structure of the NES that emerged from the EFA. The model yielded the following goodness‐of‐fit indices: *χ* ^2^/df = 3.75, RMSEA = 0.06, CFI = 0.92, NFI = 0.90, GFI = 0.90, AGFI = 0.88 (Table 3).

**TABLE 3:** Goodness of fit indicators of the NES.

Fit indices	Perfect fit	Good fit	NES value	Model fit
*χ* ^2^//df	0 ≤ *χ* ^2^/df ≤ 3.00	3 ≤ *χ* ^2^/df ≤ 5.00	3.75	Good Fit
RMSEA	0 ≤ RMSEA ≤ 0.05	0.05 ≤ RMSEA ≤ 0.08	0.06	Good Fit
NFI	0.95 ≤ NFI ≤ 1.00	0.90 ≤ NFI ≤ 0.95	0.90	Good Fit
CFI	0.95 ≤ CFI ≤ 1.00	0.90 ≤ CFI ≤ 0.95	0.92	Good Fit
GFI	0.95 ≤ GFI ≤ 1.00	0.90 ≤ GFI ≤ 0.95	0.90	Good Fit
AGFI	0.90 ≤ AGFI ≤ 1.00	0.85 ≤ AGFI ≤ 0.90	0.88	Good Fit

#### 3.2.3. Criterion‐Related Validity

Positive correlations were found between the NES Structural Empowerment subdimension and the Turkish version of the CWEQ II (*r* = 0.73) and between the NES Psychological Empowerment subdimension and the Turkish version of the PEI (*r* = 0.55, *p* < 0.001).

#### 3.2.4. Reliability Analyses

The NES Cronbach’s alpha coefficient was 0.98 for the total scale and ranged between 0.91 and 0.98 at the factor level. No item removal was found to increase the Cronbach’s alpha level. All reverse‐coded items showed a negative correlation with the total scale score, while all other items showed a positive correlation with it (*r*_max = 0.80, *r*_min = 0.40; *p* < 0.001). There was also a positive relationship between the subdimension scores of the scale (*p* < 0.001) (Table 4). A difference was found between the lower and upper 27% groups of the NES in item/factor differentiation analyses (*p* < 0.001).

**TABLE 4:** Cronbach’s *α* and intercorrelations of the NES factors.

Factors	Cronbach’s *α*	1	2	3
Structural empowerment (1)	0.98			
Behavioral empowerment (2)	0.96	0.44^∗^		
Psychological empowerment (3)	0.91	0.50^∗^	0.69^∗^	
Total empowerment	0.98	0.81	0.83	0.86

^∗^Correlation is significant at the 0.001 level.

Participating nurses in the NES test–retest analysis included 60.4% with a university degree or higher and 39.6% with a Health Vocational High School or associate’s degree level of education. By professional role, 73% were clinical nurses, 13% were nurse managers, and 14% were nurse specialists. The NES test–retest analysis showed a correlation between the two measurements of *r* = 0.86 and a correlation of *r* = 0.73–0.83 between the subdimension scores (*p* < 0.001). The intra‐class correlation coefficient value was not determined.

## 4. Discussion

The aim of the study was to develop a comprehensive measurement tool to evaluate nurses’ perceptions of empowerment. The attitudes, behaviors, and similar characteristics of individuals in various fields can be determined using scales or tests composed of purpose‐specific items; however, certain conditions must be met for such scales to be considered usable [42, 46]. Accordingly, the scale development steps recommended in the literature were rigorously followed in this study.

The empowerment concept encompasses a broad theoretical scope. Accordingly, a broad and comprehensive item pool was constructed at the initial stage, following Clark and Watson’s recommendation that initial item pools should adequately sample each main content area of the structure [56]. To avoid item redundancy or cross‐loading effects, specific methodological steps were taken during the scale development process. During item pool preparation, each item was mapped to its corresponding theoretical structure to prevent semantic overlap. Face and content validity were evaluated by a panel of experts from different fields. Any overlapping items and items with low factor loadings were removed. Through this multistage, methodologically rigorous process (encompassing face validity, content validity, and construct validity screening), item elimination was conducted in an evidence‐based manner.

EFA was used to determine the number of underlying factors and the interrelationships among items [54]. A cutoff value of 0.30 was adopted for the EFA, following the minimum threshold recommended in the literature [46, 57, 58]. Field notes that while a factor loading of 0.50 or higher indicates that a variable is well‐represented, 0.30 serves as the baseline threshold [59]. In this study, the minimum threshold for construct validity was aligned with the objective of developing the NES as a multidimensional, umbrella measure. Premature exclusion of potentially meaningful items was avoided to preserve the conceptual structure during the early stages of scale development. Accordingly, items with factor loadings between 0.30 and 0.49 were retained on the basis of their theoretical consistency with empowerment frameworks.

As a result of the analyses, in the resulting 103‐item structure of the NES, factor loadings ranged between 0.41 and 0.80. This pattern reflects the cumulative effect of the iterative screening process, which incorporated correlation‐matrix screening, communality screening, and cross‐loading removal. The concentration of loadings above the minimum threshold enhances the extent to which the retained 103 items are represented by their respective factors.

The number of factors was fixed at three based on the theoretical framework that had guided scale development since the item‐pool stage, instead of relying solely on statistical indices such as eigenvalues or scree plots. Hair et al. put forward the a priori *criterion*, according to which the number of factors to retain can be specified in advance when theoretical grounds are sufficiently strong, and this decision aligns with that criterion [51]. Fabrigar et al. [60] make a comparable point, noting that decisions about factor retention should not be left to statistical criteria alone but should also draw on existing theory. Watkins treats theoretical convergence—the alignment between the extracted structure and the conceptual model it is meant to reflect—as an accepted basis for retaining factors, stressing that each retained factor should be theoretically interpretable [61]. The three‐factor solution obtained here meets that condition: it aligns with the theoretical model that had shaped the construct from the very start and lends it empirical support, which in turn strengthens the scale’s construct validity.

The explained total variance rate of the factors determined as a result of EFA indicates to what degree the scale represents the construct it is measuring. A value of 40%–60% for the explained total variance is usually seen as adequate in the social sciences [46, 51, 57]. The variance explained by the NES was therefore interpreted as reflecting the extent to which it represents the construct of empowerment.

The model goodness‐of‐fit indices provided the desired values. These indices are used to test how much the designed model conforms to reality and therefore the structural validity of the model [62]. According to the analysis results, the ratio of the chi‐square value to the df is below 5, indicating an acceptable level of fit [52, 63]. The RMSEA value was determined to be 0.06, and this value being below the critical threshold of 0.08 indicates that the model has a good fit [64, 65]. The values of NFI, CFI, and GFI were above 0.90, meeting the acceptable fit criteria recommended in the literature [51]. Furthermore, the AGFI value being above the 0.85 threshold supports the model’s structural validity and statistical fit with the data set [66]. The lack of values within the excellent range can be explained by the scale being the first in its field and the effect of the data collection process conditions (being on call, fatigue, and other work‐related issues). These findings indicated that each of the parameters explaining the empowerment was significant and could be explained within the same structure.

Because the NES was newly developed in this study, conducting EFA first was considered a necessary step, with CFA serving as a supplementary analysis. Using the same sample for both procedures, however, is acknowledged as a limitation of the present study. In psychometric scale development, applying EFA and CFA to identical samples carries the risk of capitalizing on chance, potentially inflating model fit indices and confirming a factor structure that reflects sample‐specific characteristics rather than a generalizable pattern [67]. Ideally, an independent sample would have been used for CFA, or data would have been gathered from the same sample at a later point in time [58]. However, sample size restrictions and the field conditions encountered during data collection were not suitable for this. The use of the same sample for both EFA and CFA, similar to our study, has also been preferred in some scale‐development studies [68–72]. Nonetheless, cross‐validating with independent samples would yield stronger evidence, and this will be pursued in future work, particularly when the short‐form version of the NES is developed.

The criterion validity of an instrument is tested by its ability to provide similar results when compared with another tool for which the validity has previously been proven [46]. A strong relationship was found between the Structural Empowerment subdimension and the CWEQ‐II. There was a moderate relationship between the Psychological Empowerment subdimension and the Spreitzer’s PEI. These results indicated that the two subdimensions of the NES were consistent with these other two scales that have similar theoretical foundations. The analysis results revealed that the Structural and Psychological empowerment subdimensions of the NES had satisfactory criterion validity.

The NES demonstrated high internal consistency for both the total scale and its subdimensions, with values of 0.80 < *α* < 1.00 generally indicating high reliability and suggesting that scale items are consistent and measure the same characteristic [46, 57]. However, the high alpha values support internal consistency but may also reflect item redundancy. Streiner states that alpha values well above approximately 0.90, especially in long scales, can reflect item redundancy rather than purely superior reliability [73]. Panayides notes that a sufficient number of items in multidimensional scales can readily yield high alpha values, but that such values alone cannot indicate unidimensionality, and that excessively high values should be interpreted with caution, as they may point to scale length or item redundancy [74]. We acknowledge that the high Cronbach’s alpha value observed here may, at least in part, be attributable to the relatively large number of items comprising the scale. Furthermore, the large number of items in the NES may also pose practical implementation challenges. In the planned short‐form study, a stricter (≥ 0.50) factor‐loading threshold will be applied during item reduction, which will decrease the total number of items and thereby help mitigate the item‐count‐driven inflation of Cronbach’s alpha.

Item discrimination analyses showed that the significant difference between the upper and lower groups for mean item and factor scores was able to differentiate between those who had high and low empowerment perception for the NES [43]. Item correlation analyses revealed that all normally coded items were positively correlated with one another, while reverse‐coded items showed negative correlations with the total score, and that the internal consistency of the scale was high.

Test–retest reliability reflects the extent to which a measurement tool yields correlated results across repeated administrations over time [54, 57]. The test–retest correlation coefficients of the NES suggest that the scale demonstrates temporal stability, although this should be interpreted as correlation‐based evidence of stability rather than a full ICC‐based reliability assessment, given that ICC was not calculated in this study.

The item‐factor distribution of the NES items matched the theoretical structure that guided the scale’s development from the outset. Looking at the content of the items within each factor, together with the relevant literature, the first factor grouped together items describing characteristics of the work environment; accordingly, this factor was named the structural empowerment subdimension. It included the structural components external to employees that helped them become active, strong participants in the organization. The structural empowerment subdimension explained a considerably higher proportion of variance than the behavioral and psychological subdimensions did. This finding fits with both the item composition of the scale and the underlying logic of empowerment theory. Structural conditions such as information, resources, support, and opportunity form the foundational determinant of employees’ organizational experience [25]. Structural empowerment, in this sense, has been characterized as central to the work environment [75].

Named BE, the second factor brought together items reflecting empowered nursing behaviors: concrete, observable actions that were either part of the nursing role itself or went beyond it. This dimension appears to have received less research attention compared to its structural and psychological counterparts within the nursing empowerment literatüre. Yet it contributed meaningfully to the NES’s total explained variance, comprising 28 of the scale’s 103 items. The findings show that BE stands on its own as a distinct, measurable part of the overall construct.

The third factor consisted of items describing what nurses were feeling: this factor was made up of psychological empowerment items concerning employees’ perceptions of their organizational role and their broader perceptions of work. Psychological empowerment is inherently narrower in scope than the external, observable structural conditions and more homogeneous in content, since what is at issue here is not something that exists externally in the work environment but rather what nurses feel internally. However, psychological empowerment has been empirically shown to be a precursor to BE, mediating the relationship between organizational and individual antecedents and nurses’ empowered behaviors [76].

The correlations between the NES subdimensions and total scale scores were significant, supporting the view that empowerment is a multidimensional, interconnected concept. Spreitzer [30] describes psychological empowerment as an outcome of empowering stimuli such as work design and management style, one that in turn drives BE: proactive management shapes how employees think and, eventually, how they act. Menon [34] makes a related point, arguing that structural and motivational approaches are not truly separate but work together to produce empowerment—a conclusion also reached by a meta‐analysis that identified structural and psychological empowerment as its core building blocks [24]. This is borne out empirically: studies have found positive relationships between structural and psychological empowerment [35, 77–83], between structural and BE [33], and between psychological and BE [45, 76, 84]. What these findings point to is that empowerment cannot be understood only in terms of the psychological changes it produces in individuals; the proactive behaviors that follow, and that shape organizational outcomes, are equally important. Put simply, an employee who works in a structurally empowering environment and feels psychologically empowered is likely to see themselves as having real influence over their work and to feel a sense of ownership over it. This, in turn, is what drives nurses to take on their responsibilities proactively and act in empowered ways. It was this integrative approach that guided the development of the NES.

In addition to enabling separate evaluation of the empowerment subdimensions (structural, psychological, and behavioral), the NES’s total score also provides a general empowerment score (GES). This is related to the gap that motivated the present study: existing empowerment scales [28, 31, 34, 35, 36, 37, 38, 39] were each developed within their own respective scope. The NES offers a distinct contribution by measuring all three dimensions within the same sample, concurrently, and within a single psychometric framework‐an approach that enhances comparability across dimensions, allows the empirical relationships among subdimensions (*r* = 0.44–0.69) to be tested within a consistent framework, and enables both general and dimension‐specific assessment through a single administration. With this capacity, the NES can be used as an umbrella scale for determining the overall level of empowerment.

## 5. Limitations

The NES demonstrates strong psychometric properties, though several limitations should be acknowledged. The findings are limited to nurses employed within a single regional hospital group in Istanbul, as well as to the specific analytic techniques applied; further research testing the scale across multisite and cross‐cultural samples is therefore warranted. The 103‐item NES also serves as a comprehensive, psychometrically sound “parent form” suited to in‐depth research, yet the large number of items may prove impractical for routine clinical or managerial use given the considerable workload nurses already carry. A short‐form version is planned to address this limitation. Criterion‐related validity could not be assessed for the BE subdimension because a suitable Turkish comparator instrument was not available. The intraclass correlation coefficient was not calculated; therefore, these findings should be interpreted as correlation‐based evidence of stability rather than a full ICC‐based reliability assessment. A further methodological limitation concerns the use of the same sample for both the exploratory and CFAs; testing the scale on independent and more diverse samples is therefore recommended for future research.

### 5.1. Implications for Nursing Management

Nurse managers can utilize the NES to assess empowerment across units and pinpoint specific structural, psychological, or behavioral deficits. Because each dimension is scored separately, managers can identify which one is underperforming and target interventions accordingly. Meanwhile, the GES allows them to track whether those interventions are having an effect and to shape workforce development on that basis. At 103 items, though, the NES in its present form lends itself more to research or in‐depth organizational assessment than to everyday clinical use; the planned short form should prove a more workable option for routine practice.

## 6. Conclusions

This study developed the NES, an instrument that assesses structural, psychological, and BE concurrently within a single measure. The scale yields both subdimension scores and a GES, and this dual functionality is what sets the NES apart from existing instruments in the literature. By measuring all three dimensions within the same sample and within a coherent psychometric framework, while also providing a single total score reflecting the overall level of empowerment, the NES allows for direct cross‐dimensional comparability and makes it possible to test the empirical relationships among subdimensions directly. In practical terms, the scale can be used to identify the dimension in which an empowerment deficit is concentrated, while its GES allows a unit’s or institution’s overall level of empowerment to be summarized in a single value, supporting the evaluation of empowerment‐focused interventions and evidence‐based decision‐making in nursing management. While the 103 item NES serves as a comprehensive research instrument, its length may limit routine clinical or managerial use; a short‐form version is currently under development to address this. Future research using independent samples across diverse institutional and cultural contexts is needed to strengthen the generalizability of these findings, and supporting such research with qualitative approaches is also strongly recommended.

## Author Contributions

Fatma Cerit Soydan: conceptualization, methodology, data curation, formal analysis, investigation, writing–original draft, and writing–Review and Editing. Ayşe Nefise Bahçecik: conceptualization, methodology, supervision, validation, and writing–review and editing.

## Funding

No external funding was received for this study.

## Disclosure

The study was derived from the Ph.D. thesis of the first author.

## Conflicts of Interest

The authors declare no conflicts of interest.

## Data Availability

The data that support the findings of this study are available from the corresponding author upon reasonable request.

## References

[1] Aiken L. H. , Sloane D. M. , Bruyneel L. et al., RN4CAST Consortium, Nurse Staffing and Education and Hospital Mortality in Nine European Countries, The Lancet. (2014) 383, no. 9931, 1824–1830, 10.1016/s0140-6736(13)62631-8. PMC4035380 24581683

[2] Griffiths P. , Ball J. , Bloor K. et al., Nurse Staffing Levels, Missed Vital Signs and Mortality in Hospitals, BMJ Quality and Safety. (2018) 27, no. 1, 20–29. 30516947

[3] Bridges J. , Griffiths P. , Oliver E. , and Pickering R. M. , Hospital Nurse Staffing and Staff-Patient Interactions: An Observational Study, BMJ Quality and Safety. (2019) 28, no. 9, 706–713, 10.1136/bmjqs-2018-008948. PMC6820291 30918050

[4] Dall’Ora C. , Meredith P. , Saville C. , Jones J. , and Griffiths P. , Nurse Staffing Configurations and Nurse Absence Due to Sickness, JAMA Network Open. (2025) 8, no. 4, 10.1001/jamanetworkopen.2025.5946. PMC12015667 40261657

[5] DiNapoli J. M. , O’Flaherty D. , Musil C. , T Clavelle J. , and Fitzpatrick J. , The Relationship of Clinical Nurses’ Perceptions of Structural and Psychological Empowerment and Engagement on Their Unit, The Journal of Nursing Administration. (2016) 46, no. 2, 95–100, 10.1097/nna.0000000000000302. 26796822

[6] Visiers-Jimenez L. , Kuokkanen L. , Leino-Kilpi H. et al., Graduating Nursing Students’ Empowerment and Related Factors: Comparative Study in Six European Countries, Health Care. (2022) 10, no. 5, 10.3390/healthcare10050754. PMC9140337 35627891

[7] Friend M. L. and Sielof C. L. , Empowerment in Nursing Literature: An Update and Look to the Future, Nursing Science Quarterly. (2018) 31, no. 4, 355–361, 10.1177/0894318418792887. 30223743

[8] Armellino D. , Quinn Griffin M. T. , and Fitzpatrick J. J. , Structural Empowerment and Patient Safety Culture Among Registered Nurses Working in Adult Critical Care Units, Journal of Nursing Management. (2010) 18, no. 7, 796–803, 10.1111/j.1365-2834.2010.01130.x. 20946215

[9] Dirik H. F. , Impact of Work Environment and Empowerment on Patient Safety Culture, 2014, Dokuz Eylül University, Institute of Health Sciences, Master of Science Thesis, Izmir.

[10] Al-Dweik G. , Al Daken L. , Abusnieneh H. , and Ahmad M. , Work Related Empowerment Among Nurses: Literature Review, International Journal of Productivity and Quality Management. (2016) 19, no. 2, 168–186, 10.1504/ijpqm.2016.078885.

[11] Asif M. , Jameel A. , Hussain A. , Hwang J. , and Sahito N. , Linking Transformational Leadership With Nurse-Assessed Adverse Patient Outcomes and the Quality of Care: Assessing the Role of Job Satisfaction and Structural Empowerment, International Journal of Environmental Research and Public Health. (2019) 16, no. 13, 10.3390/ijerph16132381. PMC6651060 31277478

[12] Küçüksarı T. , The Relationships Between Personnel Empowerment with Job Performance and Nursing Care Behaviors, Istanbul University-Cerrahpasa, Institute of Graduate Studies, Nursing Management, 2020, Master of Science Thesis, Istanbul.

[13] S Laschinger H. K. , Wilk P. , Cho J. , and Greco P. , Empowerment, Engagement and Perceived Effectiveness in Nursing Work Environments: Does Experience Matter?, Journal of Nursing Management. (2009) 7, 636–646. 10.1111/j.1365-2834.2008.00907.x 19575722

[14] Ciccolini G. , Comparcini D. , and Simonetti V. , Workplace Empowerment and Nurses’ Job Satisfaction: A Systematic Literature Review, Journal of Nursing Management. (2014) 22, 855–871. 25298049 10.1111/jonm.12028

[15] Permarupan P. Y. , Al Mamun A. , Samy N. K. , Saufi R. A. , and Hayat N. , Predicting Nurses Burnout Through Quality of Work Life and Psychological Empowerment: A Study Towards Sustainable Healthcare Services in Malaysia, Sustainability. (2020) 12, 1–18, 10.3390/su12010388.35136666

[16] Özbaş A. A. and Tel H. , The Effect of a Psychological Empowerment Program Based on Psychodrama on Empowerment Perception and Burnout Levels in Oncology Nurses: Psychological Empowerment in Oncology Nurses, Palliative & Supportive Care. (2016) 14, no. 4, 393–401, 10.1017/s1478951515001121. 26466981

[17] Zhang X. , Ye H. , and Li Y. , Correlates of Structural Empowerment, Psychological Empowerment and Emotional Exhaustion Among Registered Nurses: A Meta-Analysis, Applied Nursing Research. (2018) 42, 9–16, 10.1016/j.apnr.2018.04.006. 30029721

[18] Ali A. Z. F. , Nageep S.-M. , and Hassona F. M. , The Effect of Head Nurses’ Empowerment Educational Program on Staff Nurses’ Burnout, American Journal of Nursing Research. (2018) 6, no. 6, 625–637.

[19] Şenol Çelik S. , Sarıköse S. , and Çelik Y. , Structural and Psychological Empowerment and Burnout Among Nurses: A Systematic Review and Meta‐Analysis, International Nursing Review. (2024) 7, no. 1, 189–201. 10.1111/inr.12878 37597220

[20] World Health Organization , State of the World’s Nursing 2020: Investing in Education, Jobs and Leadership, https://www.who.int/publications/i/item/9789240003279.

[21] ICN’s Call for International Nurses Day 2026: Empower Nurses to Save Lives, https://www.icn.ch/news/icns-call-international-nurses-day-2026-empower-nurses-save-lives.

[22] Ton A. I. , The Effect of Individualism-Collectivism (IC) and Trust on Workplace Empowerment, 2009, Marmara University, Institute of Social Sciences, Master of Science Thesis, Istanbul.

[23] Rao A. , The Contemporary Construction of Nurse Empowerment, Journal of Nursing Scholarship. (2012) 44, no. 4, 396–402, 10.1111/j.1547-5069.2012.01473.x. 23062275

[24] Fragkos K. C. , Makrykosta P. , and Frangos C. C. , Structural Empowerment is a Strong Predictor of Organisational Commitment in Nurses: A Systematic Review and Meta-Analysis, Journal of Advanced Nursing. (2020) 76, no. 4, 939–962, 10.1111/jan.14289. 31820471

[25] Kanter R. M. , Men and Women of the Corporation, New York: Basic Books, 1977.

[26] Conger J. A. and Kanungo R. N. , The Empowerment Process: Integrating Theory and Practice, Academy of Management Review. (1988) 13, no. 3, 471–482, 10.2307/258093.

[27] Thomas K. W. and Velthouse B. A. , Cognitive Elements of Empowerment: An Interpretive Model of Intrinsic Task Motivation, Academy of Management Review. (1990) 15, no. 4, 666–681, 10.2307/258687.

[28] Spreitzer G. M. , Social Structural Characteristics of Psychological Empowerment, Academy of Management Journal. (1996) 39, no. 2, 483–504, 10.2307/256789.

[29] Li H. , Shi Y. , Li Y. et al., Relationship Between Nurse Psychological Empowerment and Job Satisfaction: A Systematic Review and Meta-Analysis, Journal of Advanced Nursing. (2018) 74, no. 6, 1264–1277, 10.1111/jan.13549. 29473198

[30] Spreitzer G. M. , Psychological Empowerment in the Workplace: Dimensions, Measurement and Validation, Academy of Management Journal. (1995) 38, no. 5, 1442–1465, 10.2307/256865.

[31] Irvine D. , Leatt P. , Evans M. , and Barker R. , Measurement of Staff Empowerment Within Health Service Organizations, Journal of Nursing Management. (1999) 7, no. 1, 79–95, 10.1891/1061-3749.7.1.79. 10394776

[32] Suominen T. , Savikko N. , Pukka P. , Doran D.-I. , and Leino-Kilpi H. , Work Empowerment as Experienced by Head Nurse, Journal of Nursing Management. (2005) 13, no. 2, 147–153. 15720484 10.1111/j.1365-2934.2004.00523.x

[33] Trus M. , Doran D. , Martinkenas A. , Asikainen P. , and Suominen T. , Perception of Work-Related Empowerment of Nurse Managers, Journal of Research in Nursing. (2018) 23, no. 4, 317–330, 10.1177/1744987117748347. 34394438 PMC7932195

[34] Menon S. T. , Employee Empowerment: An Integrative Psychological Approach, Applied Psychology: An International Review. (2001) 50, no. 1, 153–180, 10.1111/1464-0597.00052.

[35] Laschinger H. K. S. , Finegan J. , Shamian J. , and Wilk P. , Impact of Structural and Psychological Empowerment on Job Strain in Nursing Work Settings: Expanding Kanter’ S Model, The Journal of Nursing Administration. (2001) 31, 260–272. 11388162 10.1097/00005110-200105000-00006

[36] Lake E. T. , Development of the Practice Environment Scale of the Nursing Work Index, Research in Nursing & Health. (2002) 25, no. 3, 176–188, 10.1002/nur.10032. 12015780

[37] Boudrias J. S. and Savoie A. , Les Manifestations Comportementales De L’Habilitation Au Travail: Développement D’Un Cadre Conceptuel Et D’Un Instrument De Mesure. Behavioural Empowerment at Work: Development of a Conceptual Framework and a Measurement Instrument, Psychologie du Travail et des Organisations. (2006) 12, 119–138.

[38] Roller W. K. , Measuring Perceived Empowerment in Individual Organizational Members, 1995, Florida State University, Doctoral Thesis.

[39] Kuokkanen L. , Leino-Kilpi H. , and Katajisto J. , Do Nurses Feel Empowered? Nurses’s Assessments of Their Own Qualities and Performance With Regard to Nurse Empowerment, Journal of Professional Nursing. (2002) 18, no. 6, 328–335, 10.1053/jpnu.2002.130245. 12486639

[40] Yeşilbaeş H. and Kantek F. , Nurse Empowerment Research in Turkey: Bibliometric and Content Analysis, Aydın Journal of Health. (2021) 7, no. 2, 105–121.

[41] O’Grady T. P. and Clavelle J.-T. , The Structural Framework for Nursing Professional Governance: Foundation for Empowerment, Nurse Leader. (2020) 18, no. 2, 181–189.

[42] DeVellis R. , Scale Development Theory and Applications, Trans: Totan T. Olcek Gelistirme Kuram Ve Uygulamalar. (2017) 3rd edition, Istanbul: Nobel Press.

[43] Büyüköztürk S. , Kılıç Çakmak E. , Akgün E. O. , Karadeniz S. , and Demirel F. , Eeğitimde Bilimsel Araştırma Yöntemleri, 2018, 25th edition, Ankara.

[44] Creswell J. W. and Poth C. N. , Qualitative Inquiry and Research Design: Choosing Among Five Approaches, 2018, 4th edition, CA: Sage Publications, Thousand Oaks.

[45] Boudrias J. S. , Gaudreau P. , Savoie A. , and Morin A. J. S. , Employee Empowerment: From Managerial Practices to Employees’ Behavioral Empowerment, The Leadership & Organization Development Journal. (2009) 30, no. 7, 625–638, 10.1108/01437730910991646.

[46] Alpar R. , Spor Sağlık ve Eğitim Bilimlerinden Örnekler ile Uygulamalı İstatistik Geçerlik-Güvenirlik, 2020, 6th edition, Ankara: Detay Press.

[47] Yeşilyurt S. and Çapraz C. , A Road Map for the Content Validity Used in Scale Development Studies, Erzincan University, Journal of Education Faculty. (2018) 20, no. 1, 251–264.

[48] Sürgevı̇l O. , Tolay E. , and Topoyan M. , Yapısal Güçlendirme ve Psikolojik Güçlendirme Ölçeklerinin Geçerlilik ve Güvenirlik Analizleri, Journal of Yasar University. (2013) 8, no. 31, 5371–5391.

[49] Comrey A.-L. and Lee H.-B. , A First Course in Factor Analysis, 1992, 2nd edition, Erlbaum, Hillsdale, NJ.

[50] Koyuncu İ. and Kılınç A. F. , The Use of Exploratory and Confirmatory Factor Analyses: A Document Analysis, Education in Science. (2019) 44, no. 198, 361–388, 10.15390/eb.2019.7665.

[51] F Hair J. , Black W. C. , Babin B. J. , and Anderson R. E. , Multivariate Data Analysis, 2018, 8th edition, Cengage Learning.

[52] Tabachnick B. and Fidell L. S. , Baloglu M. , Cok Degiskenli Istatistiklerin Kullanimi Using Multivariate Statistics, Trans. Nobel Akademik Yayincilik. (2015) 6th edition.

[53] Kim H. Y. , Statistical Notes for Clinical Researchers: Assessing Normal Distribution (Using Skewness and Kurtosis, Restor Dent Endod. (2013) 38, no. 1, 52–54, 10.5395/rde.2013.38.1.52. 23495371 PMC3591587

[54] Seçer I. , Psikolojik Test Geliştirme ve Uyarlama Süreci, 2015, 1st edition, Anı Press, Ankara.

[55] Sharif-Nia H. , She L. , Osborne J J. , Gorgulu O. , Khoshnavay Fomani F. , and H Goudarzian A. , Statistical Concerns, Invalid Construct Validity and Future Recommendations, Nursing Practice Today. (2024) 11, no. 1, 16–21.

[56] Clark L. A. and Watson D. , Constructing Validity: New Developments in Creating Objective, Psychological Assessment. Advance. (2019) 10.1037/pas0000626. PMC6754793 30896212

[57] Tavşancıl E. , Tutumların Ölçülmesi Ve SPSS Ile Veri Analizi, 2014, 5th edition, Nobel Academic Press, Ankara.

[58] Stefana A. , Damiani S. , Granziol U. et al., Psychological, Psychiatric, and Behavioral Sciences Measurement Scales: Best Practice Guidelines for Their Development and Validation, Frontiers in Psychology. (2025) 15, 10.3389/fpsyg.2024.1494261. PMC11798685 39916786

[59] Field A. P. , Discovering Statistics Using IBM SPSS Statistics, 2018, 5th edition, Sage, Newbury Park.

[60] Fabrigar L. R. , Wegener D. T. , MacCallum R. C. , and Strahan E. J. , Evaluating the Use of Exploratory Factor Analysis in Psychological Research, Psychological Methods. (1999) 4, no. 3, 272–299, 10.1037/1082-989x.4.3.272.

[61] Watkins M. W. , Exploratory Factor Analysis: A Guide to Best Practice, Journal of Black Psychology. (2018) 44, no. 3, 219–246, 10.1177/0095798418771807.

[62] Yaşlıoğlu M. M. , Factor Analysis and Validity in Social Sciences: Application of Exploratory and Confirmatory Factor Analyses, Istanbul University Journal of the School of Business Special issue. (2017) 46, 74–85.

[63] Wheaton B. , Muthen B. , Alwin D. F. , and Summers G. , Assessing Reliability and Stability in Panel Models, Sociological Methodology. (1977) 8, 84–136, 10.2307/270754.

[64] Browne M. W. and Cudek R. , Bollen K. A. and Long J. S. , Alternative Ways of Assessing Model Fit, Testing Structurals Equation Models, 1993, Newbury Park,CA, 136–162.

[65] Hu L. T. and Bentler P. M. , Cutoff Criteria for Fit Indexes in Covariance Structure Analysis: Conventional Criteria Versus New Alternatives, Structural Equation Modeling. (1999) 6, no. 1, 1–55, 10.1080/10705519909540118.

[66] Schermelleh-Engel K. , Moosbrugger H. , and Müller H. , Evaluating the Fit of Structural Equation Models, Methods of Psychological Research Online. (2003) 8, no. 2, 23–74.

[67] Brown T. A. , Confirmatory Factor Analysis for Applied Research, 2015, 2nd edition, The Guilford Press.

[68] Hara Y. , Asakura K. , Yamada M. , Takada N. , and Sugiyama S. , Development and Psychometric Evaluation of the Nurses Work Values Scale, Nursing Open. (2023) 10, no. 10, 6957–6971, 10.1002/nop2.1950. 37518936 PMC10495741

[69] Han K. S. , Shin J. , and Lee S.-Y. , Development of a Communication Self-Efficacy Scale for Nurses: A Psychometric Validation Study, Journal of Korean Academy of Nursing. (2025) 55, no. 2, 269–284, 10.4040/jkan.24129. 40468671

[70] Ji-yeon K. , Hyun Sun K. , Mi-jung K. , Hi-young O. , and Mi-rae J. , Development and Psychometric Evaluation of the End-of-Life Nursing Competency Scale for Clinical Nurses, Healthcare. (2024) 12. 10.3390/healthcare12161580 PMC11354138 39201139

[71] Şahin A. , Tarsuslu B. , Yılmaz A. , Kuni F. , and Durat G. , Turkish Psychometric Characteristics of the Humanistic Practice Ability of Nursing Scale: Differences by Education, Working Year, and Professional Satisfaction, BMC Nursing. (2024) 23, no. 1, 10.1186/s12912-024-02083-9. PMC11218182 38951778

[72] Kartal A. , Korkmaz Aslan G. , and Kılınç E. , Validity and Reliability of the Spiritual Health Scale-Short Form for Turkish Nursing Students, Palliative & Supportive Care. (2022) 21, no. 2, 292–300, 10.1017/s1478951521001978. 35535806

[73] Streiner D. L. , Starting at the Beginning: An Introduction to Coefficient Alpha and Internal Consistency, Journal of Personality Assessment. (2003) 80, no. 1, 99–103, 10.1207/s15327752jpa8001_18. 12584072

[74] Panayides P. , Coefficient Alpha: Interpret With Caution, Europe’s Journal of Psychology. (2013) 9, no. 4, 687–696, 10.5964/ejop.v9i4.653.

[75] Lautizi M. , Laschinger H. K. S. , and Ravazzolo S. , Workplace Empowerment, Job Satisfaction and Job Stress Among Italian Mental Health Nurses: An Exploratory Study, Journal of Nursing Management. (2009) 17, no. 4, 446–452, 10.1111/j.1365-2834.2009.00984.x. 19531144

[76] Montani F. , Courcy F. , Giorgi G. , and Boilard A. , Enhancing Nurses’ Empowerment: The Role of Supervisors’ Empowering Management Practices, Journal of Advanced Nursing. (2015) 71, no. 9, 2129–2141, 10.1111/jan.12665. 25869300

[77] DeCicco J. , Laschinger H. , and Kerr M. , Perceptions of Empowerment and Respect: Effect on Nurses Organizational Commitment in Nursing Homes, Journal of Gerontological Nursing. (2006) 32, no. 5, 49–56, 10.3928/00989134-20060501-09. 16708984

[78] Li C. , Kuo H. , Huang H. , Lo H. , and Wang H. , The Mediating Effects of Structural Empowerment on Job Satisfaction for Nurses in Long-Term Care Facilities, Journal of Nursing Management. (2013) 21, no. 3, 440–448, 10.1111/j.1365-2834.2012.01396.x. 23410197

[79] Meng L. , Liu Y. , Liu H. , Hu Y. , Yang J. , and Liu J. , Relationships Among Structural Empowerment, Psychological Empowerment, Intent to Stay and Burnout in Nursing Field in Mainland China Based on a Cross-Sectional Questionnaire Research, International Journal of Nursing Practice. (2015) 21, no. 3, 303–312, 10.1111/ijn.12279. 25521424

[80] Singh M. and Sarkar A. , Role of Psychological Empowerment in the Relationship Between Structural Empowerment, Management Research Review. (2019) 42, no. 4, 521–538, 10.1108/mrr-04-2018-0158.

[81] İspir Ö. , Hemşirelerin Güçlendirme Algıları Ve Uygulamalar Üzerinde Kontrolleri: Hemşire Ve hasta Sonuçları, 2020, Istanbul University Cerrahpasa Educational Institute of Graduate Studies, Istanbul.

[82] Blair C. , Exploring the Relationships Among New Graduate Nurses’structural Empowerment, Psychological Empowerment, Work Engagement, and Clinical Nurse Educator Leadership in Acute Care Settings, The University of Western Master thesis. (2020) Ontario.

[83] Teixeira A. C. , Nogueira A. , Nunes J. R. , Teixeria L. , and Barbieri-Figueiredo M. C. , Professional Empowerment Among Portuguese Nursing Staff: A Correlational Study, Journal of Nursing Management. (2021) 29, no. 5, 1120–1129, 10.1111/jonm.13250. 33426759

[84] Purdy N. , Laschinger H. K. S. , Finegan J. , Kerr M. , and Olivera F. , Effects of Empowerments on Nurse and Patients’Outcomes, Journal of Nursing Management. (2010) 18, 901–913. 21073564 10.1111/j.1365-2834.2010.01172.x

